# Collaborative Sensing-Aware Task Offloading and Resource Allocation for Integrated Sensing-Communication- and Computation-Enabled Internet of Vehicles (IoV)

**DOI:** 10.3390/s25030723

**Published:** 2025-01-25

**Authors:** Bangzhen Huang, Xuwei Fan, Shaolong Zheng, Ning Chen, Yifeng Zhao, Lianfen Huang, Zhibin Gao, Han-Chieh Chao

**Affiliations:** 1 School of Informatics, Xiamen University, Xiamen 361005, China; huangbz0714@stu.xmu.edu.cn (B.H.);; 2 Navigation Institute, Jimei University, Xiamen 361021, China; 3 Department of Applied Informatics, Fo Guang University, Yilan 36247, Taiwan; 4 Department of Artificial Intelligence, Tamkang University, New Taipei City 251301, Taiwan; 5 Department of Electrical Engineering, National Dong Hwa University, Hualien 974301, Taiwan; 6 Institute of Computer Science and Innovation, UCSI University, Kuala Lumpur 56000, Malaysia

**Keywords:** Collaborative Sensing-Aware Task Offloading, integrated sensing, communication, and computation, resource allocation, Internet of Vehicles

## Abstract

Integrated Sensing, Communication, and Computation (ISCC) has become a key technology driving the development of the Internet of Vehicles (IoV) by enabling real-time environmental sensing, low-latency communication, and collaborative computing. However, the increasing sensing data within the IoV leads to demands of fast data transmission in the context of limited communication resources. To address this issue, we propose a Collaborative Sensing-Aware Task Offloading (CSTO) mechanism for ISCC to reduce the sensing tasks transmission delay. We formulate a joint task offloading and communication resource allocation optimization problem to minimize the total processing delay of all vehicular sensing tasks. To solve this mixed-integer nonlinear programming (MINLP) problem, we design a two-stage iterative optimization algorithm that decomposes the original optimization problem into a task offloading subproblem and a resource allocation subproblem, which are solved iteratively. In the first stage, a Deep Reinforcement Learning algorithm is used to determine task offloading decisions based on the initial setting. In the second stage, a convex optimization algorithm is employed to allocate communication bandwidth according to the current task offloading decisions. We conduct simulation experiments by varying different crucial parameters, and the results demonstrate the superiority of our scheme over other benchmark schemes.

## 1. Introduction

The sixth-generation (6G) wireless system will evolve into an intelligent system that integrates multiple functions, including sensing, communication, and computing, dedicated to providing users with more efficient, low-latency, and highly reliable network services [1]. As one of the key technologies for 6G wireless systems, integrated sensing and communication (ISAC) enables the efficient integration of sensing and communication functions through the sharing of hardware platforms and spectrum resources [2,3]. It not only saves network resources but also improves the performance of sensing and communication in complex environments, with widespread applications in Internet of Vehicles (IoV) scenarios [4,5]. Autonomous vehicles utilize ISAC technology to sense environmental information, such as vehicles and traffic signs, and rapidly transmit these data to other vehicles, thereby reducing the risk of traffic accidents [6]. Similarly, roadside units (RSUs) can sense road conditions and share real-time data with nearby vehicles to support decision-making. Therefore, ISAC technology enhances sensing accuracy and optimizes wireless communication quality by leveraging integrated and coordinated gains, further strengthening the collaborative capabilities of the IoV [7]. Furthermore, as more sensors (e.g., cameras, LiDAR, and millimeter-wave radar) are integrated into vehicles for environmental perception, the volume of data generated per second is substantial and must be processed in real time to ensure safe driving [8]. However, the limited onboard computing resources are insufficient to meet the high computational demands. Recent research [9,10] has combined Mobile Edge Computing (MEC) with ISAC, proposing Integrated Sensing, Communication, and Computation (ISCC) technology to address the processing requirements of vehicular tasks. Computation can provide intelligent and efficient processing capabilities for autonomous vehicles, enhancing sensing and communication performance. Specifically, MEC reduces task transmission delay and minimizes task computation delay by syncing computing capabilities to wireless infrastructure close to the vehicles [11].

Although the application of ISCC-enabled IoV holds great potential, processing the massive sensing data generated by onboard sensors presents significant challenges to the communication and computing capabilities of the network [12]. For example, in the three-dimensional reconstruction process for autonomous driving, tasks typically include image acquisition, camera calibration, pose estimation, depth estimation, depth map fusion, and rendering [13]. If the depth map fusion task needs to be processed by the MEC, the original high-resolution video frames should be uploaded to the RSU. Taking an 8K resolution video with 12 bits per pixel and a frame rate of 30 fps as an example, the data volume can reach up to 11.9 Gb, which imposes extremely high demands on communication bandwidth and delay [14]. In addition, the uneven geographical distribution of vehicles may cause load imbalance on MEC across different RSUs. Specifically, when a large number of tasks are offloaded to an RSU in high-density areas, the MEC will become overloaded and prevent the provision of high-quality services to vehicles within the coverage area.

Collaborative sensing has emerged as a promising solution to address the limitations of communication resources in IoV. In fact, RSUs equipped with sensors can acquire redundant environmental data from vehicles within their coverage area [15]. When vehicles offload tasks to the RSU, only the non-redundant sensing data need to be offloaded to reduce the communication bandwidth overhead. In addition, due to the different perception perspectives of RSUs and vehicles, RSUs need to preprocess redundant data to eliminate perception discrepancies. However, performing preprocessing at the RSU further increases the computational burden on the MEC. Some studies [16,17] propose utilizing high-speed interconnections between RSUs to form a local area network (LAN), enabling collaborative computation between MECs to improve computational resource utilization efficiency. Specifically, tasks from the high-load MEC can be offloaded to the low-load MEC for processing through LAN to reduce computation delay.

While collaborative sensing can reduce communication resource overhead to some extent, several key issues remain to be addressed. In practice, the sensing range of the RSU usually does not fully overlap with that of the vehicle [18]. As a result, the sensed data obtained by the RSU and the vehicle often differ, complicating the use of collaborative sensing to improve resource utilization efficiency. Thus, accurately identifying redundant data within the overlapping sensing ranges of the RSU and the vehicle is essential. Furthermore, decisions regarding whether tasks are processed locally on the vehicle or offloaded to the RSU significantly affect task computation latency. Additionally, the allocation of communication resources by the RSU to the vehicle directly impacts task transmission delay. Therefore, making reasonable task offloading decisions and efficiently allocating communication resources are crucial to meeting the low processing latency requirements of vehicle tasks.

In this paper, we propose a joint task offloading and resource allocation scheme based on RSU and vehicles’ collaborative sensing for ISCC-enabled IoV, aiming to minimize the processing delay of all vehicle sensing tasks. The main contributions of our work are as follows:

- We construct a sensing model to demonstrate the redundant sensing data generated by collaborative sensing between RSUs and vehicles. Based on this model, we propose a Collaborative Sensing-Aware Task Offloading (CSTO) mechanism for task offloading between vehicles and RSUs. Specifically, vehicles only need to transmit the sensing data that are not redundant with the RSU during task offloading, rather than all the raw sensing data.
- We formulate an optimization problem for joint task offloading and communication resource allocation, with the goal of minimizing the processing delay of all vehicle sensing tasks while satisfying the task processing delay constraints for each vehicle. Since the optimization problem is a mixed-integer nonlinear programming (MINLP) problem, we design a two-stage iterative optimization algorithm that decomposes it into a task offloading decision subproblem and a communication resource allocation subproblem. These subproblems are solved using the Deep Q-Network (DQN) algorithm and the convex optimization algorithm, respectively. Finally, the iterative optimization of these two subproblems obtains a suboptimal solution to the original optimization problem.
- To evaluate the performance of our scheme, we conduct simulations by varying several key parameters, including task data size, available bandwidth, and computing resources. The simulation results validate the superiority of our scheme compared to other benchmark schemes.

## 2. Related Work

The application of MEC for joint task offloading and resource allocation in the IoV to enable real-time computing is gaining significant attention. The authors of [19] proposed a game-theory-based edge-to-edge collaborative computing mechanism to tackle the challenges of vehicle task offloading and communication resource allocation. In [20], the authors proposed a dynamic scheduling scheme based on genetic algorithms to prioritize the scheduling of subtasks to minimize task completion time. The authors of [21] jointly optimized task scheduling, channel allocation, and computing resources to minimize the total task processing delay for all vehicles. In [22], the authors considered scenarios with random traffic flow and dynamic network environments, using Deep Reinforcement Learning to address computation offloading and resource allocation in dynamic environments. In [23], a distributed task offloading and resource allocation algorithm is proposed for computation-intensive tasks to balance vehicle computation time and energy consumption. However, these works primarily focus on the trade-off between communication and computing resources, overlooking the impact of sensing on task offloading and resource allocation.

ISCC integrates sensing, communication, and computing functions, optimizing resource allocation in the IoV system using sensing data. The authors of [24] proposed a novel extended Kalman filter framework that uses sensing echoes to predict vehicle positions, thereby reducing communication beam tracking overhead. In [25], the authors proposed an ISCC-based solution for vehicle target detection, aiming to maximize the effectiveness of the perceived information. The authors of [26] proposed a novel directional propagation strategy driven by ISCC, enabling traffic information dissemination with service awareness and on-demand scheduling capabilities. However, these works overlook the benefits of collaborative sensing in resource allocation within the IoV. The authors of [27] proposed a collaborative sensing data fusion method based on ISCC in which vehicles compress the sensed data and upload them to the roadside unit for fusion to extend the sensing range. The authors of [28] considered collaborative sensing between RSUs and vehicles to generate the same sensing data, with vehicles only needing to upload computation instructions to reduce communication resource consumption.

However, the above studies assume that RSUs and vehicles generate identical sensing data, which is unlikely in practical environments. Therefore, this paper presents a collaborative sensing model for RSUs and vehicles and proposes a novel task offloading mechanism to enhance the utilization of communication resources.

## 3. System Model

**Figure 1:**
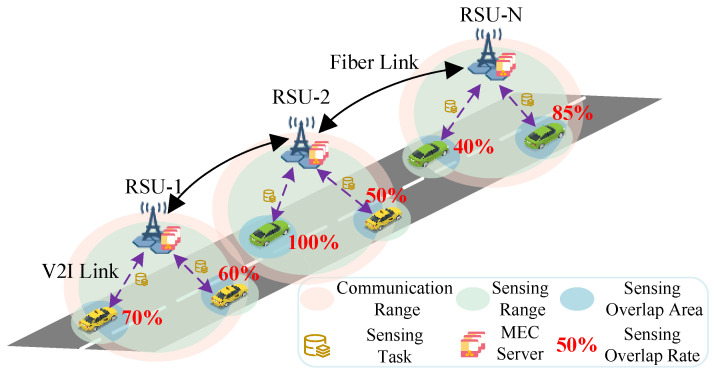
The considered ISCC system within the IoV scenario.

As shown in Figure 1, we consider an IoV scenario where the RUS and vehicles are regarded as ISCC nodes with sensing, communication, and computation capabilities. We model the road as a two-lane and unidirectional straight line with a set of RSUs N={1,2,⋯,N} deployed along it. For simplicity, we assume that each RSU covers different areas based on its sensing range. In addition, each RSU *n* (n∈N) is associated with a set of vehicles $U_{n}=\left\{1,2,\ldots ,U_{n}\right\}$ within its coverage area, and vehicle un represents the *u*-th vehicle $\left(u\in U_{n}\right)$ associated with RSU *n*. Furthermore, all RSUs equipped with mobile edge servers are interconnected via fiber optics to form a local area network (LAN) that enables mutual communication.

We assume that each vehicle un has a batch of sensing tasks (i.e., raw sensing data obtained by the sensors on the vehicle) to be processed. Specifically, the arrival of the sensing task at vehicle un follows a Poisson process with the rate $\lambda_{un}$. Further, we define the attributes of each sensing task for vehicle un as $M_{un}=\left\{d_{un},f_{un},t_{un}^{tol}\right\}$, where $d_{un}$ represents the task size, $f_{un}$ denotes the number of CPU cycles required to compute one-bit data, and $t_{un}^{tol}$ is the maximum tolerable processing delay for the task.

### 3.1. Sensing Model

Assume that RSU *n* and vehicle un are equipped with sensors featuring circular sensing ranges with radii $R_{n}$ and $R_{un}$, respectively. To define the redundant sensing data between RSU *n* and vehicle un, quad-tree compression technology is employed to partition the sensing areas into equal-area blocks, ensuring that each block contains the same amount of data [29]. Therefore, the redundant sensing data between RSU *n* and vehicle un are defined as the total area of the overlapping blocks. Additionally, considering the uncertainty in sensor performance and environmental influences [30], the sensing overlap rate $O_{un}$ between RSU *n* and vehicle un is expressed as

(1) $$O_{un}=\left\{\begin{array}{cc} 1, & \;D_{un}+r_{e}\leq R_{n}-R_{un},\\ \alpha \cdot e^{-\varphi {\left(D_{un}+r_{e}\right)}^{\phi }}, & \;\mathrm{otherwise}. \end{array}\right.$$

where $D_{un}=\sqrt{{\left(x_{un}-x_{n}\right)}^{2}+{\left(y_{un}-y_{n}\right)}^{2}}$ is the Euclidean distance between vehicle un and RSU *n*. $\alpha =\frac{S_{un}^{n}}{S_{un}}$ indicates the proportion of the intersecting area between the sensing ranges of RSU *n* and vehicle un relative to the sensing area of vehicle un. The variables $r_{e}$, φ and ϕ are determined by the characteristics of the sensors and environmental factors.

### 3.2. Proposed CSTO Mechanism

**Figure 2:**
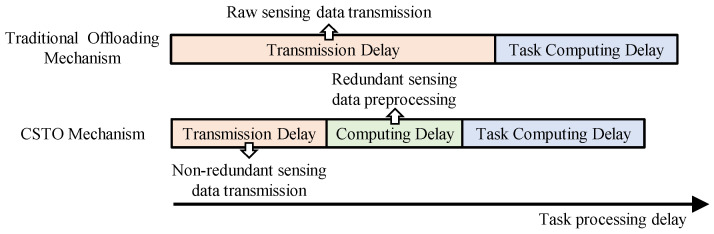
Task processing delay for different mechanisms.

To reduce task transmission delay, we propose a novel task offloading mechanism called CSTO. As depicted in Figure 2, the traditional offloading mechanism requires the vehicle to offload all raw sensing data to the RSU, which consumes significant communication resources. Due to the redundant sensing data between the vehicle and the associated RSU, the vehicle can adopt the CSTO mechanism to offload the non-redundant sensing data to the RSU. Next, considering the differing perception perspectives of the RSU and the vehicle, the RSU needs to perform coordinate transformation preprocessing on the redundant sensing data. Finally, the RSU will compute the sensing tasks. Given the small size of the computed result, its transmission delay can be ignored [31].

In this paper, we consider that the sensing tasks of the vehicle can be computed locally, offloaded to the associated RSU, or offloaded to another RSU via the associated RSU. Specifically, the vehicle sensing tasks can be computed locally to enhance the real-time performance of task processing. When the computational resources of the vehicle are insufficient, it can utilize the CSTO mechanism to offload tasks to the RSU for processing, thereby effectively reducing both transmission and computation latency. Additionally, when the computing resources of the associated RSU are insufficient, tasks can be further offloaded to another RSU for computation to reduce computation delay. To this end, we define $\eta_{un}\in \left\{0,1,2,\ldots ,N\right\}$ as the task offloading decision of the vehicle un. Here, $\eta_{un}=0$ indicates that vehicle un computes tasks locally, $\eta_{un}=n$ means that vehicle un offloads tasks to the associated RSU *n*, and $\eta_{un}=v\left(v\neq n\right)$ means that vehicle un offloads tasks to RSU *v* through the associated RSU *n*.

### 3.3. Task Processing Model

#### 3.3.1. Computing on the Vehicle

When the tasks of the vehicle un are computed locally (i.e., $\eta_{un}=0$), the tasks’ computation delay is expressed as

(2) $$t_{un}^{veh}=\frac{d_{un}f_{un}\lambda_{un}}{C_{un}},$$

where $C_{un}$ denotes the computing resource of vehicle un.

#### 3.3.2. Computing on the RSU

When the vehicle un offloads tasks to the associated RSU *n* (i.e., $\eta_{un}=n$) for processing, the task processing delay includes both computation delay and task transmission delay. Similar to [32,33], we assume that the radio access system of each RSU is based on OFDM (Orthogonal Frequency Division Multiplexing), which allocates orthogonal bandwidth resources to different vehicles, ensuring that no interference occurs during task offloading. Then, we formulate the transmission rate from vehicle un to RSU *n* is given by

(3) $$r_{un}^{v2r}=\beta_{un}B_{n}\log_{2}\left(1+\frac{p_{un}h_{un}}{\sigma^{2}}\right),$$

where $p_{un}$ is the transmission power of vehicle un and $h_{un}$ is the channel gain between vehicle un and RSU *n*. $\sigma^{2}$ denotes the noise power. Additionally, $B_{n}$ denotes the available bandwidth of RSU *n*, while $\beta_{un}$ represents the bandwidth ratio allocated to vehicle un by RSU *n*.

According to the CSTO mechanism, when the vehicle performs task offloading, it only needs to transmit the sensing data that are not redundant with the RSU *n*. Therefore, the transmission delay from the vehicle un to the RSU *n* can be expressed as

(4) $$t_{un}^{n,v2r}=\frac{\left(1-O_{un}\right)d_{un}\lambda_{un}}{r_{un}^{v2r}}.$$

Note that $O_{un}=1$ indicates complete redundancy in the sensing data between vehicle un and RSU *n*. In this case, the vehicle needs to transmit a command containing its position information to RSU *n* for coordinate transformation. Given the small size of the command, we neglect its transmission delay [28].

Considering that the task computed on the RSU undergoes a queuing process, we model the task computation process on the RSU as an M/M/1 queue. We denote $C_{n}$ as the computing resource of the RSU *n*. Then, the tasks’ computation delay from the vehicle un offloading to the RSU *n* is expressed as

(5) $$t_{un}^{n,comp}=\frac{f_{un}d_{un}\lambda_{un}+O_{un}X_{tra}d_{un}\lambda_{un}}{C_{n}-\sum_{k\in N}\sum_{l\in U_{k}}\left(f_{lk}+O_{lk}X_{tra}\right)d_{lk}\lambda_{lk}},$$

where $X_{tra}$ is the computation intensity of coordinate transformation, and $O_{un}X_{tra}d_{un}\lambda_{un}$ denotes the computation required for coordinate transformation of redundant sensing data between vehicle un and RSU *n* [14]. $f_{un}d_{un}\lambda_{un}$ denotes the computation required for the task of the vehicle un, and $\sum_{k\in N}\sum_{l\in U_{k}}\left(f_{lk}+O_{lk}X_{tra}\right)d_{lk}\lambda_{lk}$ represents the total computational load of all tasks offloaded to the RSU *n*, including tasks from associated vehicles, as well as those transmitted from other RSUs. Therefore, the processing delay for offloading tasks from vehicle un to RSU *n* is given by

(6) $$t_{un}^{n,rsu}=t_{un}^{v2r}+t_{un}^{n,comp}.$$

When RSU *n* lacks sufficient computational capacity to process the tasks of vehicle un, it can further transmit the tasks to another RSU *v* (i.e., $\eta_{un}=v,v\neq n$). Additionally, since the transmission rate of fiber-optic communication can reach 8Gbps and the task size is typically only tens of Mb, we ignore the transmission delay of vehicle tasks between RSUs [16]. Then, we denote $t_{un}^{v,comp}$ as the tasks computing the delay for offloading from vehicle un to RSU *n*, and further offloading to RSU *v*, which can be calculated based on (5). To summarize, the processing delay for offloading from the vehicle un to RSU *n* and then to RSU *v* is given by

(7) $$t_{un}^{v,rsu}=t_{un}^{v2r}+t_{un}^{v,comp}.$$

## 4. Problem Formulation

According to the system model, the task processing delay of vehicle un is defined as

(8) $$t_{un}=\left\{\begin{array}{cc} t_{un}^{veh}, & \mathrm{if}\;\eta_{un}=0,\\ t_{un}^{n,rsu}, & \mathrm{if}\;\eta_{un}=n,\\ t_{un}^{v,rsu}, & \mathrm{if}\;\eta_{un}=v,\;v\neq n. \end{array}\right.$$

According to (8), vehicles make different task offloading decisions, i.e., local computation, offloading to the associated RSU, or offloading to other RSUs via the associated RSU, resulting in varying task processing delays. Therefore, we need to find the optimal task offloading decision to minimize the task processing delay for the vehicles. Additionally, the communication resources allocated by the RSU to offloading vehicles also influence the task processing delay. Thus, it is essential to efficiently allocate the limited RSU communication resources to the vehicles requiring task offloading to reduce task transmission delay.

Let $\eta ={\left\{\eta_{un}\right\}}_{u\in U_{n},n\in N}$ and $\beta ={\left\{\beta_{un}\right\}}_{u\in U_{n},n\in N}$ denote the task offloading decision and bandwidth allocation ratio variables, respectively. The optimization problem is formulated to jointly optimize task offloading decisions and communication resource allocation to minimize the total task processing delay for all vehicles while satisfying the task processing delay constraints for each vehicle and ensuring the stability of the task queue. Thus, we construct the optimization problem as

$$\begin{array}{cc} P1: & \min_{\eta ,\beta }\sum_{n\in N}\sum_{u\in U_{n}}t_{un}\\ s.t. & C1:\eta_{un}\in \left\{0,1,2,\ldots ,N\right\},\forall u\in U_{n},\forall n\in N,\\ & C2:\beta_{un}\in \left[0,1\right],\forall u\in U_{n},\forall n\in N,\\ & C3:\sum_{u\in U_{n}}\beta_{un}\leq 1,\forall n\in N,\\ & C4:t_{un}\leq \lambda_{un}t_{un}^{tol},\forall u\in U_{n},\forall n\in N,\\ & C5:C_{n}-\sum_{k\in N}\sum_{l\in U_{k}}\left(\left(f_{lk}+O_{lk}X_{tra}\right)d_{lk}\lambda_{lk}\right)>0\\ & \;\;\;\forall l\in U_{k},\forall n,k\in N, \end{array}$$

where the constraint C1 represents the set of available task offloading decisions for the vehicle. The constraints C2–C3 ensure that the total bandwidth allocated by the RSU to associated vehicles cannot exceed its maximum available bandwidth. The constraint C4 ensures that the task processing delay does not exceed the maximum tolerable delay. The constraint C5 dictates that the computational workload of tasks processed on the RSU must be less than its computing resources to ensure the stability of the task queue.

## 5. Two-Stage Iterative Algorithm Design

P1 is a complex MINLP problem for the following reasons: (1) the variables in η are discrete, while the variables in β are continuous; and (2) the objective function and constraints C4–C5 are nonlinear. Additionally, the variables in P1 are high-dimensional and strongly coupled, posing challenges for traditional solution methods. Specifically, the communication resources allocated by the RSU to vehicles depend on different task offloading decisions. Moreover, due to the impact of resource allocation, the effectiveness of the offloading decision cannot be evaluated until the resource allocation is determined. Therefore, we design a two-stage iterative optimization algorithm to solve P1 efficiently.

In the first stage, we solve the task offloading decision (TOD) problem to determine the offloading decision for each vehicle. Since the generation of offloading decisions is a typical NP-hard optimization problem, finding the optimal decision is challenging. Deep Reinforcement Learning (DRL) combines deep learning with reinforcement learning, using neural networks to optimize task decision-making in complex environments. Therefore, we propose a DQN-based task offloading algorithm that optimizes offloading decisions for each vehicle to minimize the total task processing delay.

In the second stage, communication resources are allocated based on task offloading decisions to further reduce the overall task processing delay. Specifically, we prove that the communication resource allocation (CRA) subproblem is a convex problem and obtain its optimal closed-form solution through the Lagrange multiplier method.

Finally, the TOD and CRA subproblems are solved iteratively until the objective function value converges or the maximum number of iterations is reached, ultimately obtaining the suboptimal solution for P1.

### 5.1. Stage 1: Optimizing Task Offloading Decision

Given the bandwidth allocation ratio β, the TOD subproblem can be formulated as

$$\begin{array}{cc} & P2:\min_{\eta }\sum_{n\in N}\sum_{u\in U_{n}}t_{un}\\ & s.t.\;C1,C4,C5. \end{array}$$

Since the objective function and constraints C4 and C5 are nonlinear, P2 is a non-convex problem and cannot be solved using traditional methods. DQN is a reinforcement learning method that combines the advantages of online learning and function approximation with deep neural networks, effectively solving complex problems. Therefore, we propose a DQN-based task offloading algorithm to obtain the optimal offloading decision.

#### 5.1.1. DQN Agent

We employ the DQN agent to solve the task offloading problem, which can be formulated as a Markov Decision Process (MDP) problem as follows.

- State: The system state is defined as $st=\{un,\beta_{un}\}$, where un represents the current vehicle, and $\beta_{un}$ denotes the bandwidth ratio allocated to vehicle un by the associated RSU *n*.
- Action: The action is denoted as a={0,1,2,…,N}, representing the task offloading decision. Given the current system state, *a* = 0 denotes that vehicle un computes the tasks locally. *a* = *n* indicates that the tasks of vehicle un are offloaded to the associated RSU *n* for processing, while a=v(v≠n) means that the tasks are offloaded to RSU *v* through RSU *n* for processing.
- Reward: To achieve the optimal task offloading decision, it is crucial to design a reward function related to the objective function. Considering the limited computational resources of the vehicle, we prioritize offloading tasks to RSU with greater computational resources to minimize task processing delay. Accordingly, the reward function is defined as
  (9) $$r=\left\{\begin{array}{cc} t_{un}^{veh}-t_{un}, & s.t.\;C1,C4,C5,\\ -\mu , & \mathrm{otherwise}, \end{array}\right.$$
  where μ represents the penalty term, which is applied when constraints C1, C4, and C5 are not satisfied.

#### 5.1.2. DQN Update

DQN consists of an experience replay memory D and two deep neural networks (DNNs). The replay memory buffer ϕ is used to store observed experiences $\left(st^{l},a^{l},r^{l},st^{l+1}\right)$. The DNN includes an evaluation network and a target network, where $Q^{eval}$ and $Q^{tar}$ represent their Q-values under state $st^{l}$, respectively. These two networks are trained using experiences from the replay memory with parameter vectors ω and $\omega^{-}$, respectively.

At each step, the current state $st^{l}$ is fed into the evaluation network to obtain $Q^{eval}\left(st^{l},a^{l};\omega \right)$. The action can be selected using the ϵ-greedy algorithm, either choosing the optimal action given by $a^{l}=\arg\max_{a^{l}}Q^{eval}\left(st^{l},a^{l},\omega \right)$ or a random action with probability ϵ. After choosing the action $a^{l}$, we obtain the next state $st^{l+1}$. However, due to the queuing process in the computational model, the reward $r^{l}$ for action $a^{l}$ taken in state $st^{l}$ can only be obtained after actions have been selected for all states. These experiences are stored in the replay memory, from which random batches of data will be sampled to train the DNN. After training, the $Q^{eval}\left(st^{l},a^{l};\omega \right)$ will approach the $Q^{tar}\left(st^{l},a^{l};\omega^{-}\right)$, which is given by

(10) $$Q^{tar}\left(st^{l},a^{l};\omega^{-}\right)=r+\gamma \max_{a^{\left(l+1\right)}}Q^{\prime }\left(st^{l+1},a^{l+1};\omega^{-}\right),$$

where *r* is the reward for choosing $a^{l}$ in the state $st^{l}$, $Q^{\prime }\left(st^{l+1},a^{l+1};\omega^{-}\right)$ is used to approximate the Q-value of taking action $a^{l+1}$ in state $st^{l+1}$, and γ is the discount factor. The loss function is established according to the mean squared error (MSE) of the $Q^{eval}$ and the $Q^{tar}$, which is given by

(11) $$L\left(\omega \right)=E\left[{\left(Q^{tar}\left(st^{l},a^{l};\omega^{-}\right)-Q^{eval}\left(st^{l},a^{l};\omega \right)\right)}^{2}\right].$$

To minimize the difference between the $Q^{eval}$ and the $Q^{tar}$, the network weight parameter ω is updated using the gradient descent method, which is represented as

(12) $$\begin{array}{cc} \nabla_{\omega }L\left(\omega \right) & =E\left[(Q^{tar}\left(st^{l},a^{l};\omega^{-}\right)-Q^{eval}\left(st^{l},a^{l};\omega \right))\right]\times \nabla_{\omega }Q^{eval}\left(s^{t^{l}},a^{l};\omega \right). \end{array}$$

**Figure 3:**
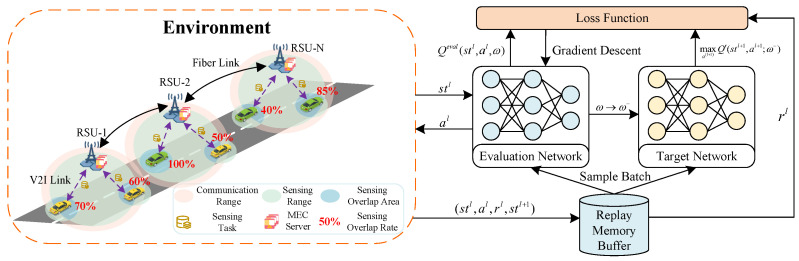
Framework of the DQN algorithm.

In addition, the initial parameters of the evaluation network and the target network are the same value. The parameters ω of the evaluation network are updated at each step, while the parameters $\omega^{-}$ of the target network are updated every fixed κ step. Figure 3 shows the detailed DQN algorithm.

In summary, the details of the proposed DQN-based task offloading algorithm are described in Algorithm 1.
**Algorithm 1** DQN-Based Task Offloading Algorithm  1:Initialize replay memory D to capacity ϕ;  2:Initialize evaluation network with random weights ω;  3:Initialize target network with weights $\omega^{-}=\omega$;  4:**for** episode=1,2,3…**do**  5:    **for** l=1,2,3… **do**  6:        Observe current system state $st^{l}$;  7:        With probability ε select random action $a^{l}$;  8:        Otherwise, select $a^{l}=\mathrm{argma}x_{a}Q^{eval}\left(st^{l},a^{l};\omega \right)$;  9:    **end for**10:    **for** l=1,2,3… **do**11:        Execute action $a^{l}$ and observe reward $r^{l}$ and $st^{l+1}$;12:        Store transition $\left(st^{l},a^{l},r^{l},st^{l+1}\right)$ in D;13:        Sample random minibatch of transitions $\left(st^{j},a^{j},r^{j},st^{j+1}\right)$ form D;14:        Set $Q^{tar}\left(st^{j+1},a^{j+1};\omega^{-}\right)=r+\gamma \max_{a^{\left(j+1\right)}}Q^{\prime }\left(st^{j+1},a^{j+1};\omega^{-}\right)$;15:        Perform gradient descent to update the network parameters ω by (12);16:        The target network parameter $\omega^{-}$ is periodically replaced by the evaluation         network parameter ω when *l* is a multiple of κ;17:    **end for**18:**end for**

### 5.2. Stage 2: Optimizing Communication Resource Allocation

According to the task offloading decisions η provided in the first stage, the CRA subproblem can be formulated as

$$\begin{array}{cc} & P3:\min_{\beta }\sum_{n\in N}\sum_{u\in U_{n}}t_{un}\\ & s.t.\;C2-C4. \end{array}$$

**Theorem** **1.** *The CRA subproblem is a convex problem.*

**Proof** **of Theorem 1.** To prove that the CRA subproblem is convex, we first describe the first derivative of the objective function with respect to $\beta_{un}$ as follows:

(13) $$\frac{\partial t_{un}\left(\beta \right)}{\partial \beta_{un}}=\frac{-\left(1-O_{un}\right)d_{un}\lambda_{un}{\left(\beta_{un}\right)}^{-2}}{B_{n}\log_{2}\left(1+\frac{p_{un}h_{un}}{\sigma^{2}}\right)}$$

Then, the second derivative can be expressed as follows:

(14) $$\frac{\partial^{2}t_{un}\left(\beta \right)}{\partial {\left(\beta_{un}\right)}^{2}}=\frac{2\left(1-O_{un}\right)d_{un}\lambda_{un}{\left(\beta_{un}\right)}^{-3}}{B_{n}\log_{2}\left(1+\frac{p_{un}h_{un}}{\sigma^{2}}\right)}$$

From (14), it follows that the second derivative is $\frac{\partial^{2}t_{un}\left(\beta \right)}{\partial {\left(\beta_{un}\right)}^{2}}>0$, proving that the objective function in the CRA subproblem is convex. Additionally, constraints C3 and C4 are linear and convex, respectively. Therefore, the CRA subproblem is convex.    □

To simplify the solution of the CRA subproblem, we define $x_{un}$ as

(15) $$x_{un}=\frac{\lambda_{un}\left(1-O_{un}\right)d_{un}}{B_{n}\log_{2}\left(1+\frac{p_{un}h_{un}}{\sigma^{2}}\right)}.$$

Since the CRA subproblem is a convex problem, we solve it using the Lagrange multiplier method to obtain the optimal closed-form solution. Specifically, we introduce non-negative Lagrange multipliers $\psi_{n}$ and $\xi_{un}$ for constraints C3 and C4. Then, the objective function is integrated with constraints C3 and C4 to form the Lagrangian function. The Lagrangian function is expressed as

(16) $$\begin{array}{cc} L(\beta ,\xi ,\psi ) & =\sum_{n\in N}\sum_{u\in U_{n}}\frac{x_{un}}{\beta_{un}}+\sum_{n\in N}\psi_{n}\left(\sum_{u\in U_{n}}\beta_{un}-1\right)\\ \; & \;+\sum_{n\in N}\sum_{u\in U_{n}}\xi_{un}\left(t_{un}-\lambda_{un}t_{un}^{tol}\right). \end{array}$$

Then, the Karush–Kuhn–Tucker (KKT) conditions can be written as

(17) $$\frac{\partial L(\beta ,\xi ,\psi )}{\partial \beta_{un}}=-\frac{x_{un}}{\beta_{un}^{2}}+\psi_{n}-\xi_{un}\frac{x_{un}}{\beta_{un}^{2}}=0,$$

(18) $$\psi_{n}\left(\sum_{u\in U_{n}}\beta_{un}-1\right)=0,\;\psi_{n}\geq 0,$$

(19) $$\xi_{un}\left(t_{un}-\lambda_{un}t_{un}^{tol}\right)=0,\;\xi_{un}\geq 0.$$

By solving Equation (17), we get

(20) $$\beta_{un}={\left(\frac{x_{un}\left(1+\xi_{un}\right)}{\psi_{n}}\right)}^{\frac{1}{2}}.$$

Therefore, we have $\psi_{n}>0$. According to (18), we obtain

(21) $$\sum_{u\in U_{n}}\beta_{un}=1.$$

Then, the optimal solutions corresponding to different values $\xi_{un}$ are summarized as follows.

When $\xi_{un}=0$, (20) can be rewritten as

(22) $$\beta_{un}={\left(\frac{x_{un}}{\psi_{n}}\right)}^{\frac{1}{2}}.$$

Substituting (22) into (21), we have

(23) $$\psi_{n}={\left(\sum_{u\in U_{n}}{\left(x_{un}\right)}^{\frac{1}{2}}\right)}^{2}.$$

Based on (21)–(23), we can obtain the optimal solution for the bandwidth allocation ratio as

(24) $$\beta_{un}=\frac{{\left(x_{un}\right)}^{\frac{1}{2}}}{\sum_{u\in U_{n}}{\left(x_{un}\right)}^{\frac{1}{2}}}.$$

When $\xi_{un}>0$, according to (19), we have $t_{un}-\lambda_{un}t_{un}^{tol}=0$. Therefore, the optimal bandwidth allocation ratio can be expressed as

(25) $$\beta_{un}=\left\{\begin{array}{ccc} \; & \frac{x_{un}}{\lambda_{un}t_{un}^{tol}-t_{un}^{n,comp}}, & \mathrm{if}\;\eta_{un}=n,\\ \; & \frac{x_{un}}{\lambda_{un}t_{un}^{tol}-t_{un}^{v,comp}}, & \mathrm{if}\;\eta_{un}=v. \end{array}\right.$$

### 5.3. Two-Stage Iterative Optimization Algorithm

Algorithm 2 describes the details of the two-stage iterative optimization algorithm, which iteratively optimizes the two subproblems until the objective value converges or the maximum number of iterations is reached, thereby obtaining the suboptimal solution for optimization problem P1.
**Algorithm 2** Two-Stage Iterative Optimization Algorithm1:Initialize i=0, $\eta^{\left(i\right)}$, $\beta^{\left(i\right)}$, the convergence threshold ρ and the maximum number of iterations *I*;2:**repeat**3:   Solve the task offloading subproblem for the given $\beta^{\left(i\right)}$ in Algorithm 1 to obtain the    solution $\eta^{\left(i+1\right)}$;4:   Solve the task offloading subproblem for the given $\eta^{\left(i+1\right)}$ to obtain the solution $\beta^{\left(i+1\right)}$;5:   Set i=i+1;6:**untilt** the objective value converges within the threshold ρ or the maximum number of iterations *I* is reached.

## 6. Performance Evaluation

In this section, we first outline the parameter settings for simulations and then evaluate the performance of our scheme by comparing it with three benchmark schemes.

### 6.1. Simulation Setting

The simulation scenario involves two high-load RSUs (RSU1 and RSU2), each associated with 10 vehicles, and one low-load RSU (RSU3) associated with 5 vehicles, reflecting the uneven geographical distribution of the vehicles. To ensure that the DQN algorithm converges more stably and efficiently to an optimal strategy, we set the discount factor γ to 0.9, the replay memory buffer ϕ to 3000, and the update frequency κ of the evaluation network parameters to 100 [34]. According to [16,28], the key simulation parameters are listed in Table 1. Additionally, the parameters $t_{un}^{tol}$, $C_{n}$, $B_{n}$, $C_{un}$, $d_{un}$, $\forall u\in U_{n},\forall n\in N$ are randomly selected from the specified ranges in the table, and the simulation results for each parameter are averaged over 30 simulations to ensure their validity and reliability.

To evaluate the performance of our scheme, we compare it with the following three schemes.

1. Average bandwidth allocation (ABA): In this approach, the RSU allocates the same bandwidth to each vehicle that needs to offload tasks [35].
2. Without collaborative computing (WCC): Tasks can be processed either locally on the vehicle or by the associated RSU [36].
3. Traditional offloading mechanism (TOM): Vehicles need to transmit raw data for task offloading.

### 6.2. Simulation Results

**Figure 4:**
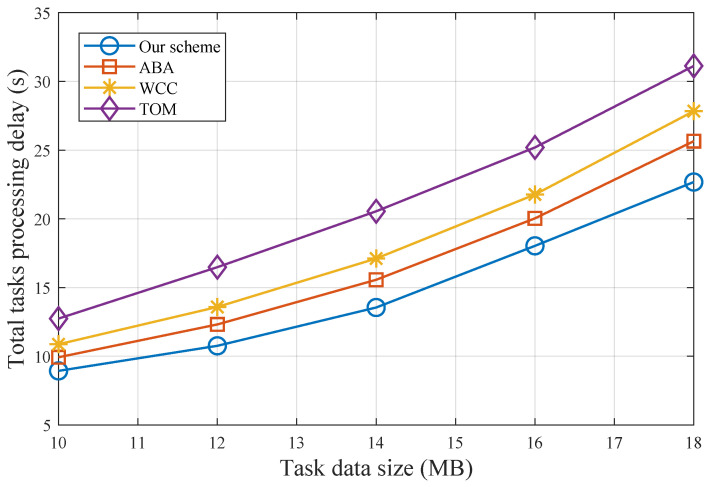
Total task processing delay with different task data sizes.

Figure 4 shows the total task processing delay for varying task data sizes. As the task data size increases, the task processing delay for TOM rises sharply due to the significant transmission delay caused by offloading raw data to the RSU. The lack of bandwidth allocation optimization and RSU collaborative computing leads to the inferior performance of ABA and WCC compared to our scheme. Notably, our scheme exhibits robustness in handling large-scale input data. For example, when the task data size is 18 MB, our scheme reduces the total task processing delay by 13.1%, 25.3%, and 37.2% compared to ABA, WCC, and TOM, respectively.

**Figure 5:**
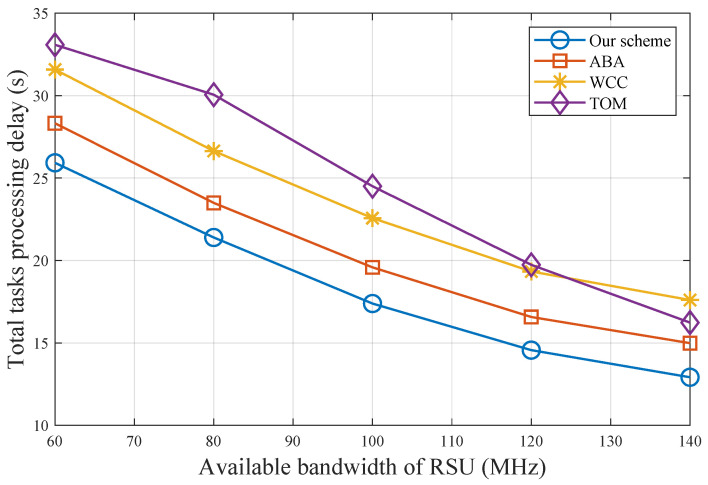
Total task processing delay with different bandwidths of RSU.

Figure 5 describes the total task processing delay for varying available bandwidth of RSU. With limited bandwidth, all schemes tend to compute tasks locally, which increases the total processing delay. Our scheme consistently outperforms others by optimizing bandwidth allocation, and more importantly, leveraging the CSTO mechanism to further reduce task transmission delay. When the available bandwidth is sufficiently large, offloading raw data to the RSU does not incur significant transmission delay, causing the performance of TOM to approach that of our scheme.

**Figure 6:**
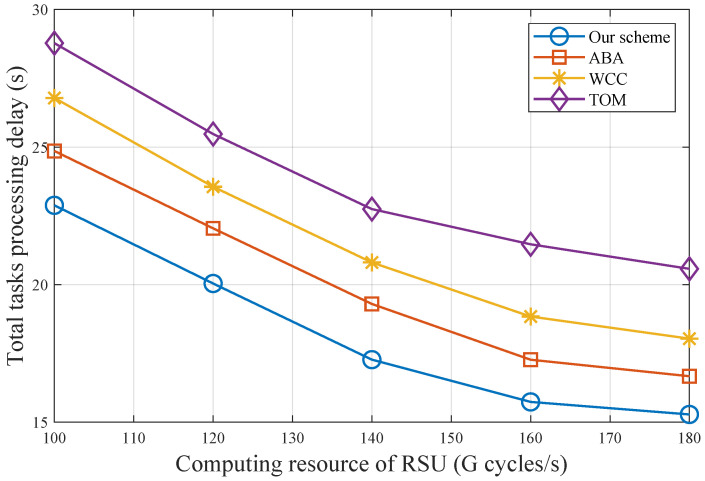
Total task processing delay with different computing resources of RSU.

Figure 6 illustrates the total task processing delay for varying computing resources of RSU. As computing resources increase, all schemes offload more tasks to the RSU, reducing processing delay. However, due to the limited available bandwidth of RSU, the rate of decrease in total tasks processing delay gradually slows. Our scheme consistently achieves the lowest task processing delay; e.g., when computing resource of RSU is 180 G cycles/s, our scheme reduces the total task processing delay by 9.4%, 18.1%, and 34.6% compared to ABA, WCC, and TOM, respectively. This demonstrates that the performance improvement of CSTO is more significant than that of TOM, when communication bandwidth becomes a bottleneck.

**Figure 7:**
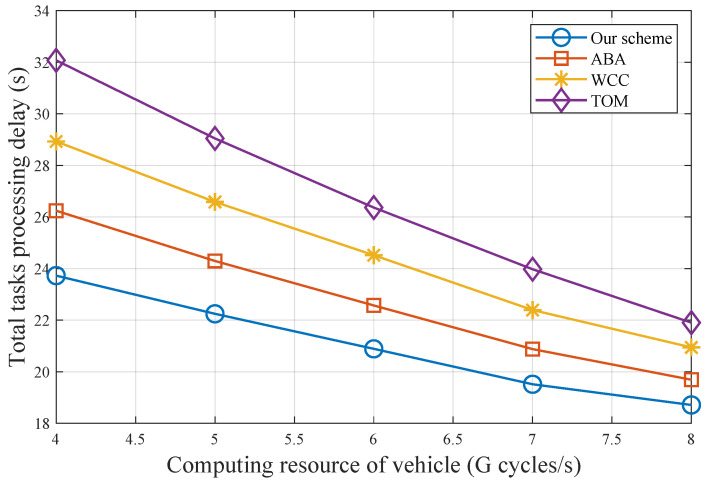
Total task processing delay with different computing resources of vehicles.

Figure 7 shows the total task processing delay for varying computational resources of vehicles. It can be observed that the total task processing delay for all schemes decreases as the computational resources of vehicles increase, with our scheme consistently outperforming the others. However, as computational resources increase, more tasks are processed locally, which gradually diminishes the advantage of our scheme.

**Figure 8:**
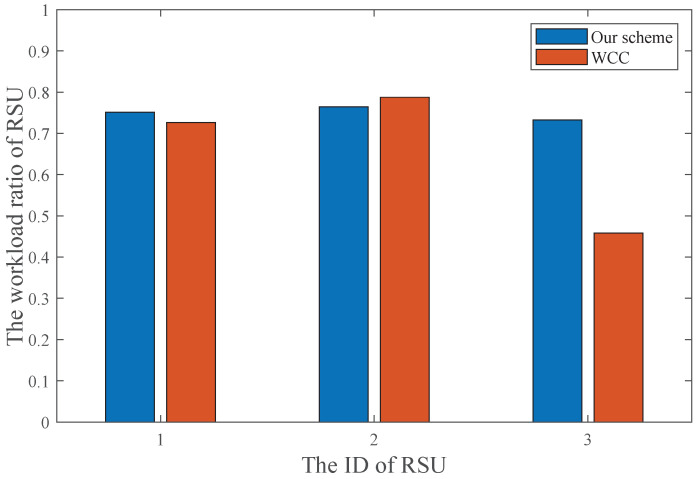
RSU workload with or without collaborative computing.

Figure 8 shows the RSU workload ratio with and without collaborative computation, where the RSU workload ratio is defined as the proportion of the required computational resources for tasks offloaded to the RSU relative to the total computational resources of the RSU. It can be observed that in our scheme, the workloads of the three RSUs are approximately equal, whereas in the WCC scheme, the workload of RSU3 is significantly lower than that of RSU1 and RSU2. The main reason is that our scheme considers RSU collaborative computing, enabling high-load RSUs (i.e., RSU1-2) to offload some sensing tasks to low-load RSU (i.e., RSU3), thereby maximizing the utilization of computing resources. In contrast, the WCC scheme lacks collaborative computing, leading to the underutilization of computing resources at RSU3.

## 7. Conclusions

In this paper, we propose a new CSTO mechanism for ISCC that allows vehicles to transmit only the sensing data that are non-redundant with the RSU during task offloading. The optimization objective is to jointly optimize task offloading decisions and communication resource allocation to minimize the processing delay of all vehicular sensing tasks. Since the optimization problem is a complex MINLP problem, it is difficult to solve using traditional methods. Therefore, we design a two-stage iterative optimization algorithm that decomposes the original problem into a task offloading decision subproblem and a communication resource allocation subproblem and solves them iteratively to obtain a suboptimal solution. The superiority of our scheme is demonstrated through simulation experiments with four different parameters and by comparing it with three baseline schemes.

In addition, our scheme may perform poorly in IoV scenarios that involve a large number of vehicles and RSUs. For example, as the number of vehicles and RSUs increases, the convergence speed and performance of the DQN algorithm may decrease in larger state and action spaces. Although convex optimization can provide the optimal solution for the communication allocation subproblem, the iterative process of the proposed two-stage algorithm may lead to significant computational overhead in real-time applications. Therefore, it is worth investigating more advanced reinforcement learning algorithms (e.g., Proximal Policy Optimization, PPO) to address complex and dynamic IoV scenarios. Furthermore, we are going to consider the impact of synchronization delays on task offloading and resource allocation.

## Figures and Tables

**Table 1:** The simulation parameters.

Parameter	Value
Transmission power of the vehicle $p_{un}$	20 dBm
Computation density $f_{un}$	100 CPU cycle/bit
Computation intensity of coordinate transformation $X_{tra}$	40 CPU cycle/bit
Noise power $\sigma^{2}$	−100 dBm
Processing delay tolerance $t_{un}^{tol}$	2–4 s
Task arrival rate $\lambda_{un}$	1 task/s
Computing resource of the RSU $C_{n}$	100–180 G CPU cycle/s
Available bandwidth of the RSU $B_{n}$	60–140 MHz
Computing resource of the vehicle $C_{un}$	4–8 G CPU cycle/s
Task data size $d_{un}$	10–18 MB
Convergence threshold ρ	0.01

## Data Availability

The data are contained within the article.
